# Mercury in Fur of Bats in Middle Taiga of the European Part of Russia at Low Anthropogenic Exposure

**DOI:** 10.3390/toxics12120863

**Published:** 2024-11-28

**Authors:** Elena Ivanova, Liubov Eltsova, Oleg Shapkin, Yuri Udodenko, Olga Rumiantseva, Yana Pevcova, Alex Viskontene, Viktor Komov

**Affiliations:** 1 Department of Biology, Cherepovets State University, 5 Lunacharsky pr., 162602 Cherepovets, Russia; esivanova@chsu.ru (E.I.); lskhabarova@chsu.ru (L.E.); udu@ibiw.ru (Y.U.); ianedakina@chsu.ru (Y.P.); vkomov@ibiw.ru (V.K.); 2 Darwin State Nature Biosphere Reserve, 162646 Borok, Russia; oshpkn@gmail.com; 3 Physiology and Toxicology Laboratory, Papanin Institute for Biology of Inland Waters, Russian Academy of Sciences, 152742 Borok, Russia; 4 Laboratory for the Study of Parasitic Arthropods, Zoological Institute, Russian Academy of Sciences, 199034 Saint Petersburg, Russia; alex.viskontene@gmail.com

**Keywords:** mercury, environmental monitoring, risk assessment

## Abstract

Mercury is considered to be one of the chemical elements posing the greatest threats to the health of most animals and can be transferred from aquatic ecosystems to terrestrial food webs. Many bat species forage above water, and their food sources include aquatic and amphibious organisms. Bats are very sensitive to the slightest changes in the environment. The objective was to determine the accumulation of mercury in the fur of insectivorous bats in summer habitats in an area with limited anthropogenic activity in the conditions of the middle taiga in the northwest European part of the Russian Federation. In the studied species, the average values of the metal’s content (μg/g) increased in the following order: *Myotis daubentonii* (3.294 ± 0.934), *Myotis dasycneme* (3.909 ± 0.543), *Vespertilio murinus* (8.011 ± 1.136), *Pipistrellus nathusii* (8.366 ± 0.546), and *Nyctalus noctula* (8.408 ± 1.386). The key factor regarding the mercury accumulation in each bat species is the foraging strategy. The mercury content in the fur of adult bats was higher than in subadults.

## 1. Introduction

Mercury is considered to be one of the chemical elements posing the greatest threats to the health of most animals [1]. This is due to the high biogeochemical mobility of organomercury compounds and their ability to accumulate in the tissues of living organisms [2,3,4]. Metal concentrations generally increase with the increasing trophic level of the organism, and the metal can be transferred from aquatic ecosystems to terrestrial food webs [5,6].

Bats are a unique group of mammals in terms of their ecological properties. European bat species have complex thermoregulatory processes, expend significant amounts of energy on flight, and consume large numbers of insects; many are also seasonal migrants, which is associated with the need to change summer and winter habitats [7]. It has been noted that bats can be convenient bioindicators of mercury in ecosystems as they are long-lived species [8,9], with a high metabolic rate in the active state, requiring the consumption of large amounts of food [10,11,12,13,14]. Many bat species forage above water, and their food sources include aquatic and amphibious organisms that are involved in the transfer of mercury from aquatic to terrestrial ecosystems. Bats are very sensitive to the slightest changes in the environment [15,16,17,18,19,20], and some bat species are currently listed as threatened or endangered [21,22]. The main attention is paid to studies of mercury accumulation by hydrobionts and fish-eating birds and mammals [23] since it is in the aquatic environment that the conditions are present for the bacterial process of the formation of the most toxic organomercury compounds [24]. As a rule, the study of mercury in the fur of bats was carried out in areas near anthropogenic and natural sources of mercury in the atmosphere [14,25,26,27]. However, elevated mercury concentrations have also been reported in bats far from the sources of this metal [28,29].

This is the first study assessing mercury accumulation regarding bats in Russia. The objective was to determine the accumulation of mercury in the fur of insectivorous bats in summer habitats in an area with limited anthropogenic activity in the conditions of the middle taiga in the northwest European part of the Russian Federation.

## 2. Materials and Methods

### 2.1. Study Area

**Figure 1:**
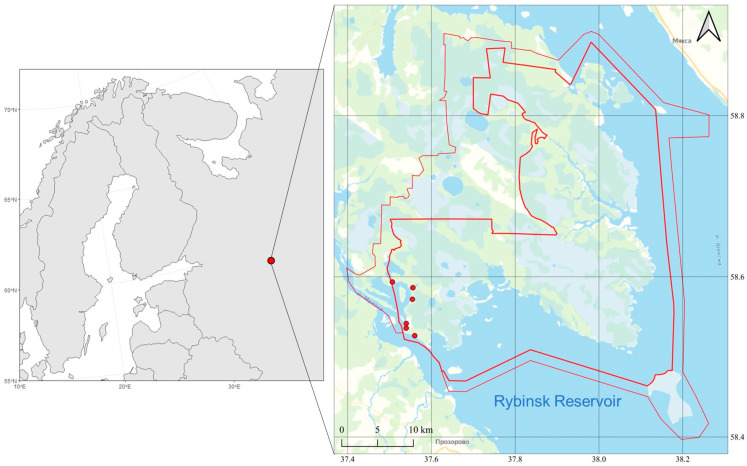
The region of study (the dots show the places where the material was collected).

Bat fur was collected in 2021–2022 in the specially protected natural area, the Darwin State Nature Biosphere Reserve, which is located on a gently sloping lowland watershed peninsula on the northwestern shore of the Rybinsk Reservoir (Figure 1).

The area of the reserve is over 112 thousand hectares, of which 67 thousand are land and the rest are coastal waters. The lowland and flat relief is dissected by a sparse network of rivers; most of the reserve is swampy.

### 2.2. Sampling

Over two years, 175 individuals of five species were studied: pond bat (*Myotis dasycneme* Boie, 1825); Daubenton’s bat (*Myotis daubentonii* Kunh, 1817); parti-colored bat (*Vespertilio murinus* L., 1758); Nathusius’s pipistrelle (*Pipistrellus nathusii* Keyserling & Blasius, 1839); and common noctule (*Nyctalus noctula*, Schreber, 1774). In 2021, bats were captured in late June. All individuals were assigned to the s/ad (subadult) age group—independent flying juvenile [30]. In 2022, material was collected in August, and all individuals were assigned to the ad (adult) age group—sexually mature individuals aged one year and older. All captured individuals were weighed to the nearest 0.1 g 8–12 h after capture. During this time, the digestive tract of bats is almost completely emptied, which reduces the weighing error since bats are able to increase their body weight by 30% during the night by eating more food [31]. The length of the forearm (R, mm) was used as the main indicator of the linear dimensions of the body. All manipulations were carried out in accordance with the recommendations of the American Society of Mammalogists [32]. The animal study protocol was approved by the Ethics Committee of the Zoological Institute RAS (1-11/21-03-2024). The bats’ fur was collected from their backs (0.1–0.2, g). The animals were kept in cloth bags. After all manipulations, bats were released into the wild the following day after sunset. The animals were checked for dehydration by assessing the elasticity of the skin; some of them were administered water to drink before release. Based on the features of foraging behavior, species of the Vespertidae family in the European part of the Russian Federation are divided into three groups [33]. The first group consists of species with high dietary plasticity, employing different hunting strategies and regularly changing the biotopes in which they feed. Among the studied species of the Darwin Nature Reserve, only the common noctule bat belongs to this group. The second group consists of conditionally plastic species; their representatives in one region hunt in different biotopes but at the same time prefer not to leave the hunting place during the night. In the Darwin Nature Reserve, this group includes Nathusius’s pipistrelle and the parti-colored bat. The third group are conservative species preferring the same food items regardless of the region. In the Darwin Nature Reserve, these are the pond bat and Daubenton’s bat.

### 2.3. Analytical Methodology

The mercury content in the fur was determined at the Regional Center for Collective Use of Cherepovets State University. The analysis was performed by the pyrolysis method on an atomic absorption spectrometer RA-915M with a PIRO attachment (the limit of detection for mercury is 0.002–200 µg/g). Fur samples weighing 10–50 mg were placed on a quartz dispenser and transferred to a thermolysis cell to determine the total mercury content. The samples were burned at a temperature of 300 °C for 1–2 min. The accuracy of the analysis was determined using certified biological material DORM-4 (0.412 ± 0.036 µg/g) and DOLT-5 (0.44 ± 0.18 µg/g) (Institute of Environmental Chemistry, Ottawa, ON, Canada). The accuracy was checked every 20 measurements (relative percentage difference (RPD) < 10%). The differences between replicates averaged 7.3%. The limit of detection (LOD), the limit of quantitation (LOQ), trueness, and precision followed the EURACHEM criteria. The LOD and LOQ values calculated using DORM-4 and DOLT-5 were 0.0004 and 0.0014 µg/g and 0.0007 and 0.0216 µg/g, respectively.

### 2.4. Statistical Analysis

Mercury concentrations in bat fur did not follow a normal distribution (Shapiro–Wilk test and Kolmogorov–Smirnov test), so nonparametric methods were used in statistical analysis: Kruskal–Wallis U-test and Mann–Whitney H-test. Spearman’s nonparametric correlation coefficient was used to determine the correlation between mercury concentration in the fur and animal’s weight.

## 3. Results

**Figure 2:**
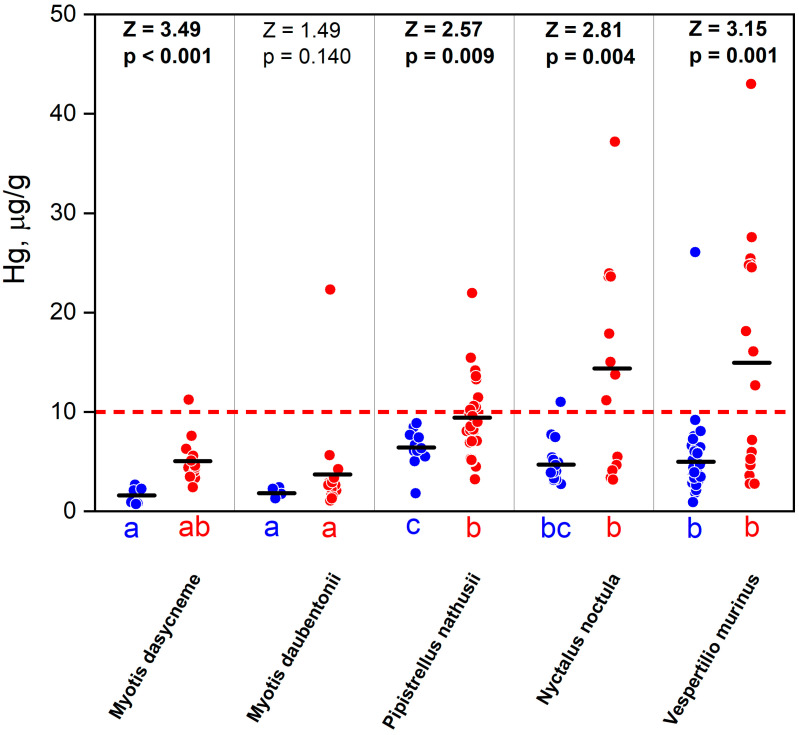
The mercury content in the fur of different bat species, where blue dots are subadults and red are adults. Under the top axis, results of Mann–Whitney tests are shown. Different letters (a,b,c) under bottom axis indicate significant differences between bat species in multiple comparisons of mean ranks (Kruskal–Wallis ANOVA). Red line is a level of risk of the sublethal neurochemical effects of mercury exposure [26].

The absolute mercury concentrations in the fur of the bats from the Darwin Nature Reserve varied widely, from 0.720 µg/g in *Myotis daubentonii* to 43 µg/g in *Vespertilio murinus*. The minimum statistically significant mercury values in the fur were noted for *Myotis daubentonii* (3.294 ± 0.934), *Myotis dasycneme* (3.909 ± 0.543), intermediate for *Vespertilio murinus* (8.011 ± 1.136), and maximum for *Pipistrellus nathusii* (8.366 ± 0.546) and *Nyctalus noctula* (8.408 ± 1.386). The same trend was noted when comparing both the young and adult individuals of the studied species separately (Table 1; Figure 2).

In all the species, the mercury content in the fur of the adult bats was 1.5–3 times higher than that of the subadults (Figure 2).

Statistically significant differences in the mercury concentrations in the fur between bats of different ages were noted in all the species, except for Daubenton’s bat species. The levels of mercury accumulation in the fur between the different species differed statistically significantly in both the juveniles and adults (H = 36.86 and H = 35.91, respectively; *p* < 0.001). The minimum average concentrations were always observed in *Myotis daubentonii* and *M. dasycneme* (in juveniles—1.610 ± 0.800 and 1.835 ± 0.482 µg/g; in adults—4.865 ± 2.191 and 3.723 ± 4.925 µg/g, respectively). The highest concentrations of mercury in the fur of the juveniles were observed in *Pipistrellus nathusii* (6.421 ± 1.700 µg/g) and in the adults in the common noctule and *Vespertilio murinus* (13.455 ± 10.144 µg/g and 14.959 ± 11.791 µg/g, respectively). The differences between the average concentration of mercury in the fur of the juveniles and adults of *Pipistrellus nathusii* are 1.5 times and in the species of the genera *Myotis* and *Vespertilio murinus* 3–5 times.

No statistically significant differences in the concentration of mercury in the fur were found between the males and females in any of the studied bat species (Table 2).

**Figure 3:**
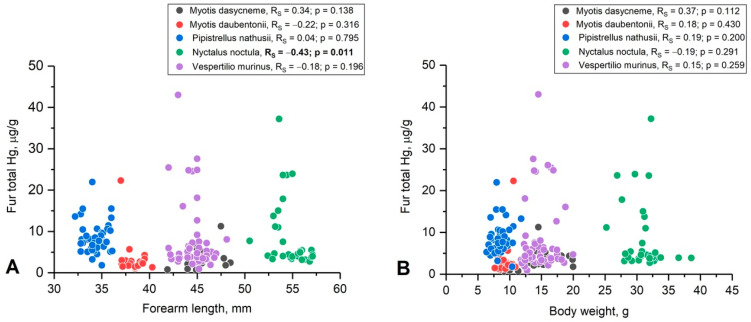
Relationship between THg in fur and body size parameters (**A**—weight and **B**—forearm length) in bats from the Darwin Nature Reserve. Spearman correlation (R_S_) and *p*-value (*p*) are provided in brackets.

Despite the fact that the studied species differ in size, no significant correlations were found between the mercury content in the fur of the bats and their size characteristics (weight and forearm length). The correlation between the mercury levels in the fur and forearm length was noted only for *Nyctalus noctule* (Figure 3).

The accumulation of mercury in some species is consistent with the characteristics of their foraging strategy. Minimum mercury concentrations were observed in conservative species, and maximum concentrations were found in those species characterized by trophic plasticity.

## 4. Discussion

The mercury content in the organs and tissues of bats depends on its background concentrations in the breeding sites and along the migration routes, including the presence of point anthropogenic mercury sources [26,34,35]. The mercury concentrations in the fur of all the bat species from the Darwin Nature Reserve are within the range observed in representatives of the Vespertidae family inhabiting areas with minimal anthropogenic stress in Europe and North America. Thus, in *Myotis daubentonii* from the Darwin Nature Reserve, the average mercury content in the fur was 2 times higher than in individuals of the same species from the southern regions of Sweden [36]. The average mercury content in the fur of adult *M. myotis* from non-industrial regions of the Czech Republic was comparable to the mercury content in the fur of adult *M. daubentonii* and *M. dasycneme* from the Darwin Nature Reserve [28]. The average mercury concentration in the fur of some Vespertilionidae species from the northeastern regions of the USA was either comparable (*Myotis leibii* and *Myotis sodalis*) or 1.5–2 times higher (*Perimyotis subflavus, Myotis lucifugus,* and *Myotis septentrionalis*) compared to *Nyctalus noctula* and *Vespertilio murinus* from the Darwin Nature Reserve, which had the highest average mercury concentrations in their fur [34].

The fur of bats from areas subject to anthropogenic influence may exhibit mercury concentrations exceeding those found in the bats from the Darwin Nature Reserve. For instance, the average mercury content in the fur of *M. myotis* from the Czech Republic, living in areas of active coal mining and industrial enterprises, was 30 µg/g, i.e., 10 times higher than that of the conspecifics from areas with low anthropogenic impact [28]. In *Myotis lucifugus* caught in the vicinity of the mercury-contaminated South River in Virginia, the average mercury concentration in the fur was 132 ± 96 µg/g, which is 10 times higher than the maximum average values recorded in adult individuals of *Nyctalus noctula* and *Vespertilio murinus* in the Darwin Nature Reserve [26]. The Darwin Nature Reserve is located at a distance from large industrial sources of mercury. As studies show, the mercury emissions from the metallurgical and chemical enterprises in the city of Cherepovets, located 70 km north of the reserve, are minimal and do not lead to increased mercury concentrations in the adjacent ecosystems [37,38,39].

Sex, age, reproductive status, and species characteristics are the most significant factors determining the level of mercury accumulation in bats in an area with a limited anthropogenic impact [34,40]. The ratio of the mercury concentrations in the fur between the age groups of the bats from the Darwin Nature Reserve is consistent with the results of other studies. For most species, it was shown that adults contain several times more mercury than juveniles [27,34,41,42]. The mercury concentrations in the fur of the bats from the temperate zone reflect the levels of its intake into the organism during the active phase of the annual life cycle, when the fur grows [43]. Therefore, the mercury concentration in the fur is an integral indicator of the mercury accumulation in the organism between seasonal molts. For a correct interpretation of the obtained results, it is necessary to consider whether the fur was taken for analysis “before” or “after” the seasonal molt. Subadult bats are a special group in which the complete replacement of the fur formed in utero occurs during the first year of life [44]. Therefore, the mercury content in the fur of juveniles reflects its intake during embryonic development and with maternal milk, and during independent feeding. The fur from the adult bats in the Darwin Nature Reserve was collected in early August, while most temperate species molt mainly in late summer–autumn. Therefore, the mercury concentration in the fur of the adult bats from the Darwin Nature Reserve reflects interspecific differences in mercury accumulation due to both its intake at breeding sites and during seasonal migrations. In addition, the increase in the mercury concentrations in the fur of the adults, compared to the juveniles, can be explained by the long-term accumulation of mercury in the organism with a decrease in the synthesis of mercury-binding proteins with age, such as metallothioneins, involved in mercury excretion.

Studies of other Vespertilionidae species show that mercury accumulation can differ statistically significantly between the sexes [34]. The average mercury content in the fur of females is generally lower than that of males, which is explained by its excretion from the body during pregnancy and lactation [45]. In addition, sex differences in mercury accumulation in bats may be associated with the fact that individuals of different sexes prefer different biotopes for feeding and wintering, and their choice is dependent on the river network density and altitude above sea level [46]. The absence of sexual differences in subadults can be explained by the fact that all the individuals of this group have the same reproductive status. The bats of this group have not yet undergone physiological changes leading to the elimination of mercury from the organism (changes in hormonal levels, pregnancy, and lactation in females).

Food is the main source of mercury for most vertebrates. The predatory species occupying the upper trophic levels in local food webs contain more mercury in their organs and tissues compared to the herbivorous species of the low trophic levels. This pattern is also true for bats with different trophic specializations. Thus, when studying 32 tropical bat species belonging to eight trophic guilds, it was shown that the minimum concentrations of mercury in the fur are observed in frugivorous species, while the concentration of mercury in the fur of insectivores was the highest [25]. Similar differences between bats of different trophic guilds were found in a study in Belize [47]. 

The results of the present study show that interspecific differences are observed not only in species from different trophic guilds but also for species belonging to the same trophic guild but preferring different food items [40,48]. The diet composition of most of the bats in the Darwin Nature Reserve has not been studied, with the exception of Nathusius’s pipistrelle [49]. However, it can be assumed that their diet is based on the same food items as among the representatives of these species from the other regions of the European part of Russia—primarily insects of different taxonomic and ecological groups, including amphibious ones [50]. Aquatic ecosystems are one of the main sources of mercury in terrestrial food webs, and the level of mercury accumulation in bat fur reflects the link of their diet with aquatic ecosystems [47]. However, despite the fact that *Myotis daubentonii* and *M. dasycneme* prefer to feed over water, studies of the trophic niches in the different bat species in the Volga region have shown that 60–70% of their diet includes butterfly and caddisfly imago [50]. These two species of bats have conservative feeding behavior, so it should be expected that their diets differ little in different parts of the range [33]. At the same time, beetles comprise a significant share of the diets of the other studied bat species. 

In addition to the composition of the diet, the rate of accumulation in the body can be influenced by the body size and physiological characteristics of individual species. Smaller species are expected to accumulate toxins faster because they have higher specific metabolism and therefore must consume prey at a higher rate. In general, mammals of different ecological and size groups do not show significant correlations between mercury content in fur and size–weight characteristics. 

The mercury content in bat fur closely correlates with its content in the skin, muscle, blood, and internal organs [28,34,35]. Therefore, fur is convenient for assessing the overall toxic impact of mercury on bat organisms.

Previous studies on mammals have established concentrations of mercury in fur leading to toxic effects. 

**Figure 4:**
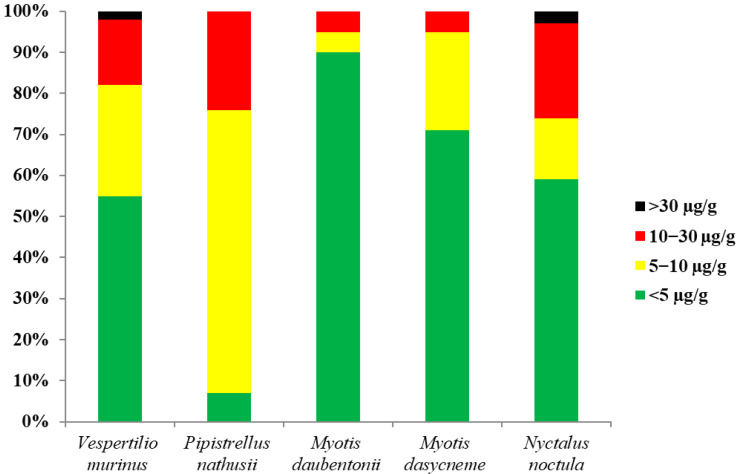
The proportion of studied bats with different mercury content in their fur.

The neurotoxic effect of the metal is primarily manifested by damage to the organs of the central nervous system [51,52,53]. Visual impairment and changes in movement activity in wild bat populations have been noted at mercury levels in fur > 5 μg/g [54]. The mercury concentrations in the fur of bats in northwestern Russia exceed 5 μg/g in 48% of the individuals studied (Figure 4).

It has been established that 10 μgHg/g in bat fur is the threshold level of risk of the sublethal neurochemical effects of mercury exposure [26,34,55]. Among the studied bats, the proportion of individuals with mercury concentrations in their fur above 10 mg/kg is 18%. Exceeding the threshold value was noted in all the studied species (Figure 4).

The threshold level of mercury content in bat fur, exceeding which increases the risk of DNA damage, is 30 μg/g [56]. Mercury content in fur exceeding 30 μg/g was found in single individuals of *Nyctalus noctula* and *Vespertilio murinus*. There is also a risk of reproductive damage: oxidative stress has been reported in the testes, for example, in rats after mercury exposure [57,58]. The effects of mercury also include changes in fetal development, which can cause impairment or even death after birth [59].

## 5. Conclusions

The study showed that 18% of the insectivorous bats had mercury concentrations above the risk threshold for the sublethal neurochemical effects of mercury exposure.

The key factor for mercury accumulation in each bat species is the foraging strategy. In addition, in all the studies of bat species, the mercury content in the fur of the adult bats was higher than that of the subadults. The size of the individual and sex do not affect mercury accumulation. The interspecies differences are due to dietary habits. Future studies should therefore focus on analyzing the element concentrations in prey insects foraged by different bat species.

## Figures and Tables

**Table 1:** Multiple Comparisons *p* values, Kruskal-Wallis test of different bat species.

	*Myotis dasycneme*	*Myotis daubentonii*	*Pipistrellus nathusii*	*Nyctalus noctula*	*Vespertilio murinus*
subadult H (4; 85) = 36.9, *p* < 0.001					
*Myotis dasycneme*	-	1.000	**<0.001**	**0.007**	**0.004**
*Myotis daubentonii*		-	**<0.001**	**0.040**	**0.032**
*Pipistrellus nathusii*			-	0.124	**0.028**
*Nyctalus noctula*				-	1.000
*Vespertilio murinus*					-
adult H (4; 91) = 35.9, *p* < 0.001					
*Myotis dasycneme*	-	1.000	0.077	0.122	0.056
*Myotis daubentonii*		-	<0.001	**<0.001**	**<0.001**
*Pipistrellus nathusii*			-	1.000	1.000
*Nyctalus noctula*				-	1.000
*Vespertilio murinus*					-

In bold, significant differences between bat species are shown.

**Table 2:** Weight, forearm length, and THg concentrations in fur of male and female bats from the Darwin Nature Reserve by species. In M–W columns, Mann–Whitney test results are displayed.

Species	Sex	Weight, g	M–W	Forearm Length, mm	M–W	THg	M–W
*Myotis dasycneme*	male n = 12	13.2 ± 3.2 8.1–20.0	**Z = 1.99** ***p* = 0.046**	44.4 ± 3.0 36.2–48.0	Z = 0.78 *p* = 0.432	3.310 ± 2.969 0.720–11.240	Z = 1.89 *p* = 0.057
	female n = 8	15.0 ± 2.6 9.9–19.4		45.1 ± 3.1 37.8–48.5		4.350 ± 1.037 2.425–5.569	
*Myotis daubentonii*	male n = 5	8.7 ± 1.3 7.7–10.9	Z = 1.70 *p* = 0.090	38.5 ± 0.8 37.2–39.5	Z = 1.31 *p* = 0.185	1.964 ± 1.291 1.072–4.232	Z = 1.49 *p* = 0.140
	female n = 17	9.5 ± 0.6 8.5–10.6		38.3 ± 0.9 37.0–40.3		3.685 ± 4.903 1.299–22.310	
*Pipistrellus nathusii*	male n = 18	8.0 ± 1.1 6.4–10.4	Z = 1.46 *p* = 0.145	33.8 ± 0.9 32.2–35.0	**Z = 2.91** ***p* = 0.036**	8.789 ± 4.807 1.799–21.950	Z = 0.012 *p* = 1.0
	female n = 26	8.6 ± 1.2 6.7–11.8		35.5 ± 3.1 33.0–46.0		8.134 ± 2.762 3.205–15.490	
*Nyctalus noctula*	male n = 11	30.5 ± 2.3 25.2–33.4	Z = 1.89 *p* = 0.057	54.5 ± 1.5 52.4–57.1	Z = 1.89 *p* = 0.057	7.988 ± 9.965 2.736–37.200	Z = 0.21 *p* = 0.836
	female n = 22	31.1 ± 2.8 26.9–38.6		54.2 ± 4.7 34.0–57.1		8.651 ± 7.427 3.146–23.930	
*Vespertilio murinus*	male n = 19	13.8 ± 1.5 12.0–17.5	**Z = 2.10** ***p* = 0.036**	44.4 ± 1.3 42.0–46.8	Z = 1.14 *p* = 0.251	4.324 ± 1.694 0.929–7.547	Z = 1.87 *p* = 0.06
	female n = 37	15.3 ± 3.0 11.8–27.4		44.9 ± 1.3 42.0–48.1		9.904 ± 9.905 1.882–43.005	

Note: above the line mean value ± standard deviation, below the line - minimum and maximum values. In bold, significant differences between male and female bats species are shown.

## Data Availability

The data presented in this study are available on request from the corresponding author.
